# Effect of tobacco smoke on hydrogen sulfide-induced rat thoracic aorta relaxation

**DOI:** 10.1590/1414-431X20165592

**Published:** 2017-02-06

**Authors:** H.T. Zhang, T. Zhang, M. Chai, J.J. Sun, X.Y. Yu, C.Z. Liu, C.C. Huang

**Affiliations:** 1 Department of Cardiology, Air Force General Hospital of the Chinese People’s Liberation Army, Beijing, China; 2 Department of Cardiology, The First People’s Hospital of Chuzhou, Chuzhou, China; 3 Department of Cardiology, Lung and Blood Vessel Disease, Ministry of Education, Beijing Anzhen Hospital, Capital Medical University, Beijing Institute of Heart, Beijing, China

**Keywords:** Tobacco smoke, Hydrogen sulfide, Cardiac smooth muscle, Cystathionine-γ-lyase, Sulfonylurea receptor-2

## Abstract

Levels of hydrogen sulfide (H_2_S), a gaseous signaling molecule, are reduced in the serum of individuals who smoke. We hypothesized that tobacco smoke influenced smooth muscle relaxation by decreasing H_2_S levels and this effect could also influence expression of cystathionine γ-lyase (CSE) and sulfonylurea receptor-2 (SUR-2). The aim of this study was to explore the effect of tobacco smoke on H_2_S-mediated rat thoracic aorta relaxation and its possible mechanism. Thirty-two Sprague-Dawley rats were divided into four groups: control (C) group, short-term smoker (SS) group, mid-term smoker (MS) group, and long-term smoker (LS) group. H_2_S concentrations in serum, action of H_2_S on rat aortic vascular relaxation, and expression of CSE and SUR-2 in thoracic aortic smooth muscle were measured. Although there was no significant difference in H_2_S between the C and the SS groups, concentration of H_2_S was significantly reduced in both the LS and MS groups compared to control (P<0.01). Furthermore, H_2_S was significantly lower in the LS than in the MS group (P<0.05). Rat aortic vascular relaxation was lower in all three treatment groups compared to the control, with the most significant decrease observed in the LS group (P<0.05 compared to the MS group). Expression of CSE and SUR-2 was reduced in the LS and MS groups compared to control (P<0.05), with the lowest levels observed in the LS group (P<0.05). Therefore, tobacco smoke reduced expression of CSE and SUR-2 in rat thoracic aorta, which may inhibit H_2_S production and vascular dilation.

## Introduction

Smoking is of great harm to human health. According to the World Health Organization tobacco epidemiology report of 2011, smoking causes approximately 6 million deaths every year, with about a third of these deaths resulting from heart or cerebrovascular complications (1). Epidemiological studies provide evidence that smoking is highly associated with cardiovascular disease, a major risk factor for hypertension and coronary heart disease (1). Although tobacco combustion produces over 500 types of harmful substances, some of the most damaging to the human body include carbon monoxide (CO), nicotine, and tar. Additionally, long-term smoking generates reactive oxygen species and free radicals that promote oxidative stress and damage to the vascular intima layer and blood vessels (2). In addition to these well-known harmful effects of smoking, cigarette smoke also significantly reduces levels of hydrogen sulfide (H_2_S) in the serum (3).

H_2_S is one of the three gases, including nitric oxide (NO) and CO that function as signaling molecules in the body (4). H_2_S has important signaling and regulatory roles in multiple tissues throughout the body, including the mammalian central nervous system, the cardiovascular system, the digestive system, and the urogenital system (5,6). H_2_S is naturally synthesized in the body from L-cysteine through the enzymatic action of cystathionine-beta synthase (CBS) and cystathionine γ-lyase (CSE); of these, CSE is the enzyme predominantly expressed in the cardiovascular system (7,8). H_2_S regulates the function of the K_ATP_ channel located in vascular smooth muscle cell membranes, by hyperpolarizing the cell membrane, inhibiting the internal flow of Ca^2+^ and causing vascular smooth muscle relaxation (9,10). The K_ATP_ channel is comprised of four inwardly rectified potassium channel subunits (Kir) (11), of which two subtypes are present in mammals, Kir6.1 and Kir6.2. While Kir6.1 is predominantly expressed in the heart, coronary artery smooth muscle cells and endothelial cells, Kir6.2 is mainly expressed in ventricular muscle cells and endothelial cells (11). The K_ATP_ channel is activated by H_2_S in combination with the sulfonylurea receptor (SUR), which has two subtypes – SUR-1 and SUR-2. The SUR-2 subtype is primarily expressed in myocardial cells and vascular smooth muscle of the cardiovascular system (12).

Many earlier studies have focused on the effect of tobacco smoke on the NO-induced relaxation of cardiovascular and pulmonary vascular smooth muscle (13). In contrast, reports describing the impact of H_2_S on relaxation of vascular smooth muscle are limited to the pulmonary and mesenteric arteries and the portal vein. Moreover, the conclusions from these studies are not entirely consistent and appear to be dependent on the type of vessels being investigated (14–18). Despite inconsistencies, H_2_S is vitally important to proper heart function. H_2_S relaxes vascular smooth muscle, reduces blood pressure, and accelerates apoptosis and inhibits proliferation of vascular smooth muscle cells (19). H_2_S can also expand the coronary artery, increase myocardial blood flow, reduce injury induced by myocardial ischemia, inhibit cytochrome C oxidase, reduce oxidative phosphorylation of tissues and organs, reduce the consumption of ATP, protect myocardial function, and reduce myocardial cell apoptosis (20).

Based on previous findings, we hypothesize that tobacco smoke influences smooth muscle relaxation by, at least in part, decreasing levels of H_2_S. Altered concentration of H_2_S may also influence expression of CSE and SUR-2. In this study, we investigated the effect of tobacco smoke on H_2_S-mediated rat thoracic aorta relaxation and its possible mechanism of action.

## Material and Methods

### Establishment of animal model

The study was approved by the animal care review committee of Air Force General Hospital. Thirty-two male Sprague-Dawley rats, weighing between 200∼250 g, were obtained via the Academy of Military Sciences Laboratory Animal Center (manufacturing license No.: SCXK-J-2007-004). Rats were randomly divided into four groups, with 8 rats in each group. These included the control group (C): raised for 90 days with normal breathing; the short-term smoking group (SS): exposed to 20 cigarettes (Hong Mei, Hongta Group, China, smoke nicotine content: 1.1 mg, tar: 12 mg, flue gas CO: 13 mg) per day for 30 consecutive days; the mid-term smoking group (MS): exposed to 20 cigarettes per day for 60 consecutive days, and the long-term smoking group (LS): exposed to 20 cigarettes per day for 90 consecutive days. A homemade semi-closed organic glass box was constructed as previously described (21). Rats in the smoking groups were placed in the box, cigarettes were lit, and tobacco smoke was blown into the box by an air pump. Five cigarettes were lit each time. After a cigarette burned out, the pump continued for an additional 10 min and another cigarette was lit. In total, 20 cigarettes were lit every day.

### Determination of H_2_S in serum

Rats were anesthetized by intraperitoneal injection of 5% sodium pentobarbital at a dose of 40 mg/kg. Blood was collected from the abdominal aorta and then centrifuged at 3000 *g* at 4°C for 10 min. Serum concentration of H_2_S was measured with a sensitive sulfur electrode (Ag2/s, Shanghai ray Magnetic Instrument Factory, China). H_2_S typically exists as two forms in serum; one-third exists as H_2_S gas and two-thirds exist as sodium sulfide (NaHS). Rat serum was mixed with an isometric oxidation solution (NaOH: 8 g, EDTA: 7 g, deionized water: 85 mL, and 10 g ascorbic acid added just before use). S^2-^ ions, generated by the reaction between H_2_S and NaHS with the oxidation solution, were activated by deionized water for more than 2 h before the sulfur electrode and the reference electrode were immersed into the serum. The sulfur ion content was then determined with a PHS-25 ion meter (Shanghai Ray Magnetic Instrument Factory) and compared to the values from standard sulfur ions diluted to 1, 10, 20, 40, and 80 μM in the oxidation solution. After measuring each sample, the electrode was immersed in deionized water to maintain its active state.

### Determination of the relaxation rate of thoracic aortic rings *in vitro*

The chest cavity of each rat was opened, and the thoracic aorta was placed in Krebs liquid (NaCl: 118.3 mM; KCl: 4.7 mM; NaHCO_3_: 25 mM; Mg_2_SO_4_: 1.2 mM; KH_2_PO4: 1.2 mM; CaCl_2_: 2.5 mM; EDTA: 0.026 mM; Glucose: 11.1 mM; pH=7.4) at 4°C in a 95%-O_2_:5%-CO_2_ gas mixture. The perivascular adipose connective tissue was separated from the 3∼4 mm proximal tissue and used for *in vitro* experiments. The separated thoracic aorta segment had its vascular endothelial function damaged with a cotton swab, then placed in a constant temperature bath at 37°C in 10 mL Krebs liquid with continuous ventilation in a 95%-O_2_:5%-CO_2_ mixture. The vascular rings were connected with two metal hooks attached to the bottom of the bath; a tension sensor was also included to measure vascular tension, which was recorded with a Powlab four lead physiological recorder. The vascular static tension was maintained at 1 g, and the Krebs liquid was changed every 15 min over the course of 1 h. To ensure that the blood vessels were in a good physiological state, they were allowed to contract three times in advance by adding 68 mM KCl solution. To test endothelial function, 10^-6^ mM acetylcholine was added; if the relaxation rate was >30%, it suggested that the endothelium had not been completely removed from the vascular ring, which was discarded from the experiment. The concentration of acetylcholine used was the most effective at relaxing aorta as determined by a concentration response curve from a preliminary study. When the stable state of contraction was 100%, 10^-7^ mM phenylephrine was added. The maximum diastolic amplitude was measured after adding 10^-8^, 10^-7^, 5×10^-7^, 10^-6^, 5×10^-6^, and 10^-5^ mol/L NaHS as previously described (12). The diastolic ratio of H_2_S = (diastolic amplitude/maximum contraction amplitude) × 100%.

### Immunohistochemical detection of CSE and SUR protein expression

Thoracic aorta specimens were fixed in 10% formaldehyde and embedded in paraffin. Paraffin blocks were sliced to a thickness of 5 μm and were baked for 2 h in a 60°C oven. After dewaxing (xylene immersion for 10 min, followed by the replacement of the xylene and an additional soak of 10 min) and hydration (sequential 5 min washes in an ethanol gradient, from anhydrous, to 95%, to 70% ethanol), sections were washed three times, 5 min each, with PBS (NaCl: 137 mM, KCl: 2.7 mM, Na_2_HPO_4_: 4.3 mM, KH_2_PO_4_: 1.4 mM, pH 7.2∼7.4). Next, 3% H_2_O_2_ was added for 10 min at room temperature to stop endogenous peroxidase activity. Sections were then washed twice with PBS. Antigen retrieval was performed using a pressure cooker. Samples were then washed three times with PBS and blocked with 10% goat serum for 20 min at room temperature. CSE mouse monoclonal antibody (Abgent, USA) or SUR-2 rabbit polyclonal antibody (Santa Cruz, USA) (both primary antibodies diluted 1:200) were incubated at 37°C for 1 h. Sections were then washed three times with PBS prior to the addition of suitable secondary antibodies at 37°C for 1 h. After secondary antibody incubation, samples were once again washed three times with PBS. Next, 3,3′-diaminobenzidine chromogenic stain was added for 3 min and then rinsed off with distilled water to terminate the reaction. Detection of a brown color was taken as positive staining. As a negative control, PBS was added instead of CSE and SUR-2 primary antibodies. Five sections of one tissue were randomly inspected 400 times, and the computer color magic image analysis system (CMIAS) was used to quantitatively analyze the expression of CSE and SUR-2 proteins by measuring the area density (area density = area of positive hits/total area ×100) (21).

### Statistical analysis

Data were analyzed using SPSS 17.0 statistical software (SPSS Inc., USA). Data are reported as means±SD. For comparison among multiple groups, single factor ANOVA was used. For comparison between two groups, single factor variance of the LSD method was used. P<0.05 was considered to be statistically significant.

## Results

### H_2_S serum concentration decreased with increased exposure to cigarette smoke

**Figure 1:**
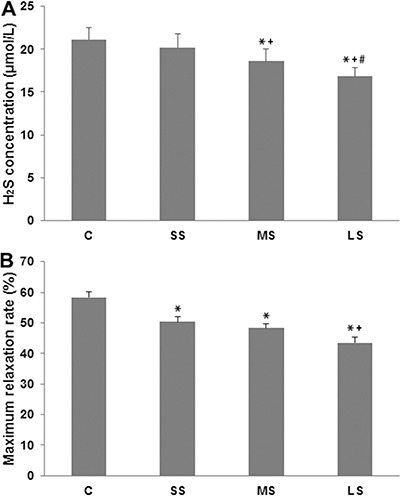
Serum H_2_S concentration (*A*) and maximum relaxation rate to NaHS of thoracic aorta (*B*) in control (C), short-term smoking (SS), middle-term smoking (MS) and long-term smoking (LS) groups. Data are reported as means±SD. *P<0.01 compared with C group; ^+^P<0.01 compared with SS group; ^#^P<0.05 compared with MS group (ANOVA).

We determined the effect of length of exposure to cigarette smoke on the serum concentration of H_2_S in rats. Serum concentration of H_2_S in the SS group (20.13±1.13 μmol/L) was not different from the control C group (20.88±1.25 μmol/L; P>0.05; Figure 1A). In contrast, serum concentrations of H_2_S were significantly reduced in both the MS group (18.25±1.04 μmol/L) and in the LS group (16.75±1.28 μmol/L; P<0.01 compared to the control group in each case). Moreover, levels of H_2_S were significantly reduced in rats in the LS group (P<0.05 compared to animals in the MS group). Taken together, we found significantly reduced serum levels of H_2_S with increased exposure to cigarette smoke over time.

### Increased exposure to cigarette smoke decreased thoracic aorta relaxation rate *in vitro*

We measured the effect of cigarette smoke exposure on the maximum diastolic rate of rat thoracic aorta. As shown in Figure 1B, short-term (50.50±6.59%; P<0.05), mid-term (47.51±5.93%; P<0.01), and long-term exposure (42.34±6.24%; P<0.01) all significantly reduced aortic relaxation rate to NaHS compared to controls (57.80±6.61%). Importantly, the relaxation rate was highly reduced in the LS group (P<0.05 compared to the SS group), suggesting that the length of cigarette smoke exposure is an important determinant of effect on thoracic aorta relaxation.

### CSE expression in rat thoracic aorta decreased with increased exposure to cigarette smoke

**Figure 2:**
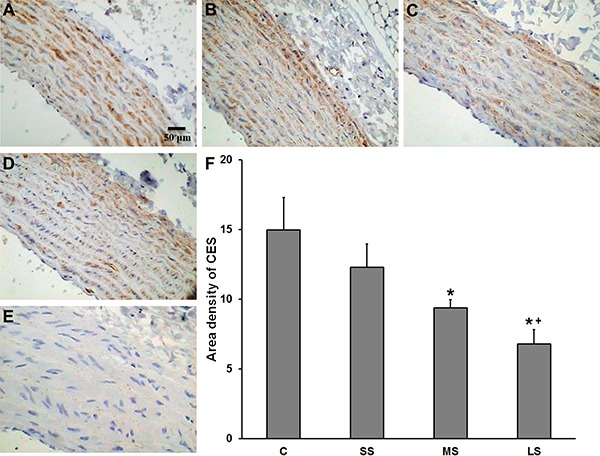
Immunohistochemical staining of cystathionine γ-lyase (CSE) expression. *A*, Control (C); *B,* short-term smoking (SS) group; *C*, middle-term smoking (MS); *D*, long-term smoking (LS) groups; *E*, negative control (×400); *F*, quantification of the staining. Data are reported as means±SD. *P<0.05 compared with C group; ^+^P<0.05 compared with SS group (ANOVA).

We performed immunohistochemistry for detection of CSE in rat thoracic aorta following exposure to cigarette smoke for different lengths of time (Figure 2A-E). We found decreased staining intensity with increased lengths of exposure, shown in Figure 2F. There was no significant difference in CSE staining intensity between the control (14.98±2.32 area density) and SS (12.30±1.68 area density) groups. However, CSE expression was significantly reduced in both the MS group (9.37±0.59 area density; P<0.05) and the LS group (6.79±1.04 area density; P<0.01) compared to control. We also detected a significant decrease in CSE expression when comparing the LS and MS groups (P<0.05). Taken together, the results showed that CSE expression decreased in rat thoracic aorta with increased exposure to cigarette smoke.

### SUR-2 expression in rat thoracic aorta decreased with increased exposure to cigarette smoke

**Figure 3:**
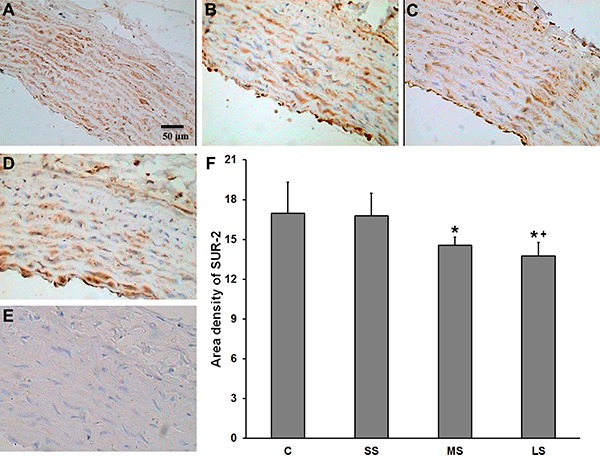
Immunohistochemical staining of sulfonylurea receptor-2 (SUR-2). *A*, Control (C); *B,* short-term smoking (SS) group; *C*, middle-term smoking (MS); *D*, long-term smoking (LS) groups; *E*, negative control (×400); *F*, quantification of the staining. Data are reported as means±SD. *P<0.05 compared with C group; ^+^P<0.05 compared with SS group (ANOVA).

We performed immunohistochemistry for detection of SUR-2 in rat thoracic aorta following exposure to cigarette smoke for different lengths of time (Figure 3A-E). We found decreased staining intensity with increased lengths of exposure (Figure 3F). There was no significant difference in SUR-2 staining between the control (16.98±1.80 area density) and SS (16.79±1.63 area density) groups. However, SUR-2 expression was significantly reduced in both the MS group (14.57±1.46 area density; P<0.05) and the LS group (13.74±1.94 area density; P<0.01) compared to control. We also detected a significant decrease in SUR-2 expression when comparing the LS and MS groups (P<0.05). Taken together, the results showed that SUR-2 expression decreased in rat thoracic aorta with increased exposure to cigarette smoke.

## Discussion

Cigarette smoke is extremely detrimental to human health. In addition to generating free radicals that can damage tissues of the cardiovascular system, smoking is also associated with decreased serum levels of H_2_S, a gaseous signaling molecule. Although H_2_S regulates smooth muscle cell relaxation, the exact mechanism of its action is unknown. In this study, we explored the effect of tobacco smoke on H_2_S-mediated rat thoracic aorta relaxation and its possible mechanism of action. We successfully established a rat model of passive cigarette smoke inhalation, and found that increased smoke exposure is associated with decreased levels of H_2_S and decreased thoracic aorta relaxation rate. We also found decreased expression of CSE and SUR-2, proteins involved in H_2_S synthesis and action, in thoracic aortas from rats exposed to smoke for long periods of time. We propose that decreased expression of these factors may, at least in part, contribute to the effect of H_2_S on vascular smooth muscle relaxation.

Our findings are consistent with an earlier report showing reduced levels of H_2_S in smokers compared to nonsmokers in an investigation in patients with chronic obstructive pulmonary disease (3). We propose that the reduced expression of H_2_S may, at least in part, be due to deceased expression of CSE, a key enzyme regulating H_2_S production in vascular smooth muscle and endothelial cells. It is important to note that the activity of CSE is not the only means of generating H_2_S within the cell. CBS also generates H_2_S; however, this enzyme predominantly functions within the central nervous system (22). Additionally, previous work has shown that 3-mercaptopyruvate sulfurtransferase in combination with cysteine aminotransferase can generate H_2_S from cysteine (23). Similar to CBS, however, these enzymes function largely in the nervous system. H_2_S production via these diverse enzymes may contribute to the overall pool of H_2_S *in vivo* and should be further examined in future work.

In this study, we also detected decreased vascular relaxation rate of the thoracic aorta with increased exposure to cigarette smoke. This occurred concomitantly with decreased H_2_S concentration, suggesting, but not proving, a causal relationship between the two. Other studies performed in different pulmonary and systemic arteries have also reported on the effect of H_2_S on relaxation and contraction. However, many have conflicting results, with H_2_S affecting relaxation and contraction in different manners. One important point to take from these studies is that the effect of H_2_S may be largely dependent on the exact concentration of H_2_S in some vessels, the type of vessel being evaluated, and the species from which the vessels are isolated (14–18,24). In our study, the decrease in thoracic aorta relaxation with increased exposure to cigarette smoke may be related to decreased expression of SUR-2, which regulates the ability of H_2_S to induce vascular smooth muscle relaxation. This finding is supported by previous work showing that H_2_S upregulates levels of both SUR-2B and Kir6.1 in vascular smooth muscle cells of hypertensive rats (25). We and others have shown that H_2_S concentrations are approximately 10^-6^∼10^-3^ mol/L in diastolic vascular smooth muscle, and that this is independent on serum concentration (26). Therefore, the effect of tobacco smoke on cardiovascular relaxation is unlikely to be directly due to the serum concentration of H_2_S. Since tobacco smoke reduces CSE expression and, subsequently, H_2_S generation in vascular smooth muscle, it may also influence cardiovascular relaxation. Moreover, since SUR-2 is a major player in signaling via the K_ATP_ channel, and since cigarette smoke exposure decreases SUR-2 expression, it may represent an additional mechanism by which cigarette smoke contributes to reduced thoracic aorta relaxation. Although interesting, additional research is necessary to understand the mechanism by which cigarette smoke actually decreases expression of CSE and SUR-2.

Tobacco smoke decreased expression of CSE and SUR-2 in vascular smooth muscle. This may, at least in part, explain the association between cigarette smoke and decreased serum concentration of H_2_S. In conclusion, we provide evidence that smoking reduced H_2_S-mediated rat thoracic aorta relaxation.
